# Supplementary material for: Microfabricated electrodes unravel the role of interfaces in multicomponent copper-based CO2 reduction catalysts
Source: Nat Commun. 2018 Apr 16;9:1477. doi: 10.1038/s41467-018-03980-9 (PMC5902587; doi:10.1038/s41467-018-03980-9)
Supplement: Supplementary file 1 — Supplementary Information [file 41467_2018_3980_MOESM1_ESM.pdf]

Supplementary Information

Microfabricated electrodes unravel the role of interfaces in multicomponent copper-based CO_2 reduction catalysts

Larrazábal et al.

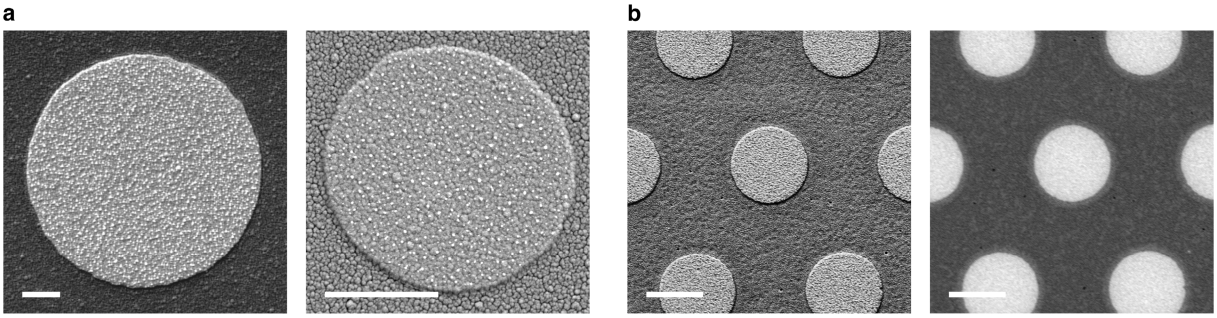

Supplementary Figure 1 | SEM micrographs from fresh and used electrodes. a, Micrographs obtained in secondary electron mode of (left) In_2O_3/Cu_2O (d = 12.5 µm) and (right) In_2O_3/Cu (d = 5 µm) fresh electrodes showing the dense structure of the layers composed of aggregated nanoscale crystallites. Scale bars: 2 µm. **b**, Micrographs obtained in secondary (left) and backscattered electron modes (right) of the same area from a used In_2O_3/Cu_2O electrode (d = 12.5 µm) showing the preservation of the geometry and distribution of the two phases, the latter evidenced by the Z-contrast in the right image. Scale bars: 10 µm.

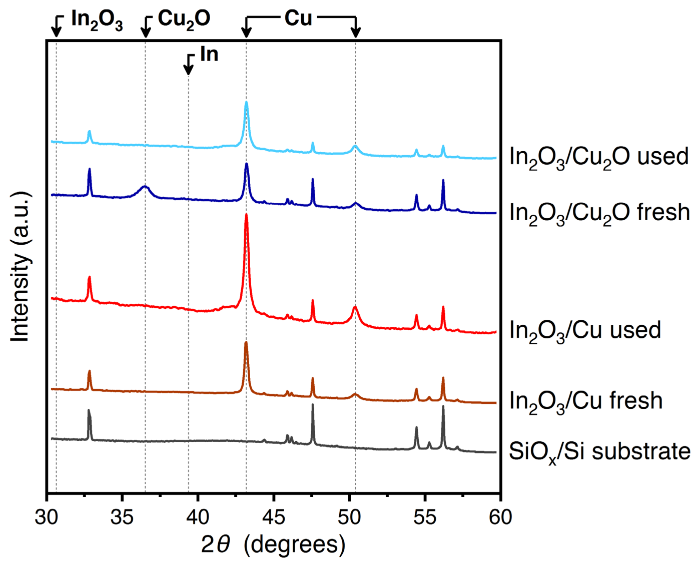

Supplementary Figure 2 | X-Ray diffractograms of selected electrodes. XRD patterns indicate the expected presence of Cu and Cu_2O in the fresh samples in Cu_2O-based electrodes (Cu_2O surfaces were sputtered on a Cu layer) and the reduction of Cu_2O to Cu after the electrolysis at −0.6 V vs. RHE for 5 min. Reflections corresponding to indium-containing species were absent likely due to their amorphous nature and/or their low concentration. The signal obtained from the underlying SiO_x/Si substrate is added for reference.

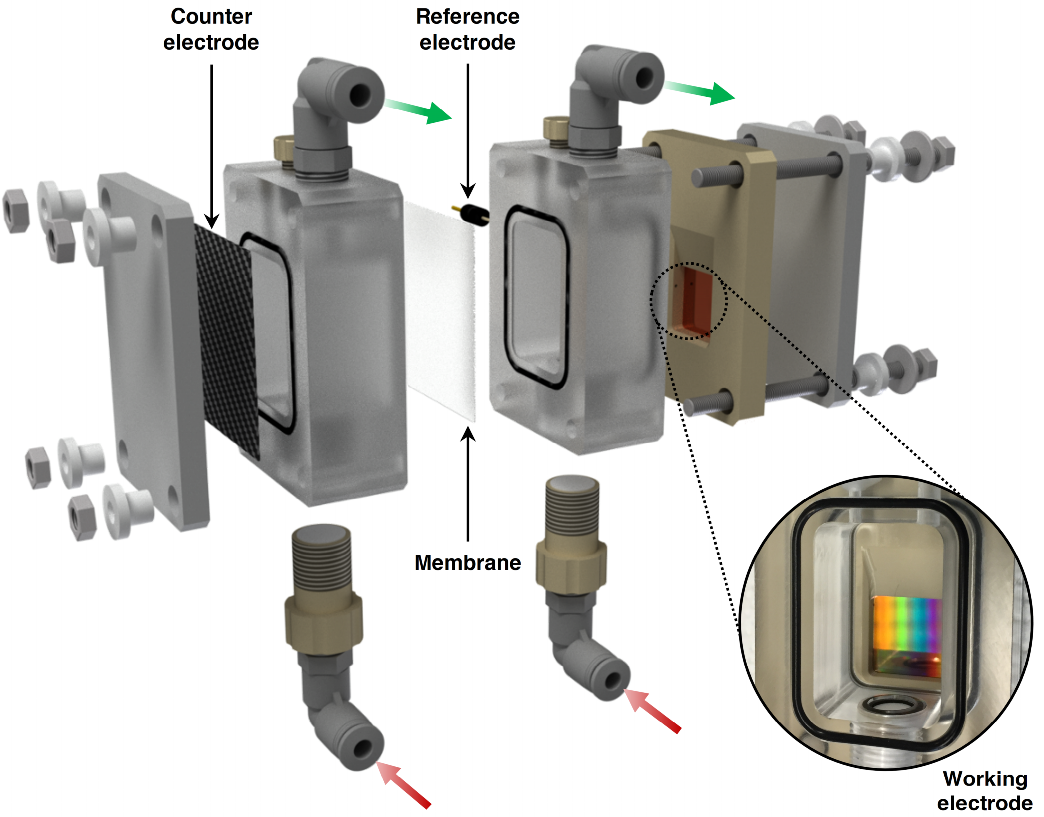

Supplementary Figure 3 | Electrochemical cell for catalytic testing. Exploded view of the custom gastight sandwich-type cell used for testing the structured electrodes under standard eCO_2RR conditions (−0.6 V vs. RHE). The cathodic and anodic compartments hold a volume of 9.5 and 7.8 cm^3 of electrolyte, respectively, and have a headspace of ca. 2 cm^3. The inlet and outlet gas streams of each compartment (20 cm^3 STP min^{-1}) are indicated by the bold red and green arrows, respectively. The outlet of the cathodic chamber was analysed by on-line gas chromatography. The inset shows a photograph of a structured In_2O_3/Cu_2O electrode (with a geometric active area of 2.25 cm^2) assembled into the cathodic compartment. A capillary defined the sensing point of the reference electrode at ca. 1 mm from the electrode surface, resulting in low and consistent values of the uncompensated resistance (ca. 12 Ω) in each experiment. Other details of the experimental setup are provided in the Methods section of the main text.

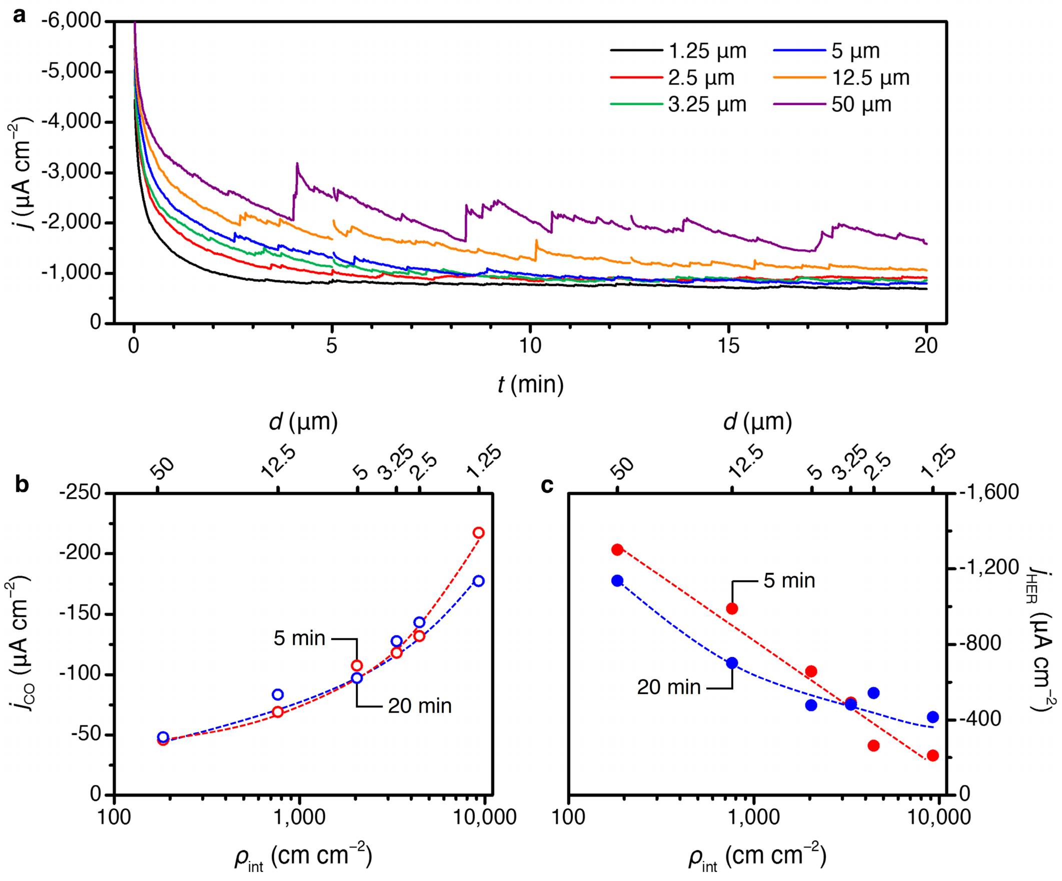

Supplementary Figure 4 | Chronoamperometric curves over structured In_2O_3/Cu_2O electrodes and catalytic activity for CO and H_2 evolution at different times. a, Total current density (j) as a function of time (t) in 20-minute CO_2 reduction electrolyses at −0.6 V vs. RHE over electrodes with different island sizes. In all experiments, the current decays rapidly during the initial 3 minutes of the electrolysis due to the reduction of the In_2O_3 islands and of the Cu_2O substrate. Thereafter, the current generally shows a more stable behavior with a slightly decreasing trend. Jumps in the current density are caused by the detachment of bubbles formed on the surface of the electrodes (particularly with large island sizes). The uncompensated resistance was re-measured and updated 5 and 12.5 min after the start of each electrolysis. Partial current density for **b**, CO (j_{CO}) and for **c**, H_2 (j_{HER}) as a function of the interfacial density (ρ_{int}) over In_2O_3/Cu_2O electrodes at 5 and 20 min following the start of the eCO_2RR electrolysis. The corresponding island diameter (d) is also indicated and the trendlines are added as a visual aid. Both data sets show similar trends, indicating the suitability of the 5-minute tests to assess the catalytic behaviour of the electrodes and suggesting a rapid equilibration of the headspace of the cathodic compartment of the cell.

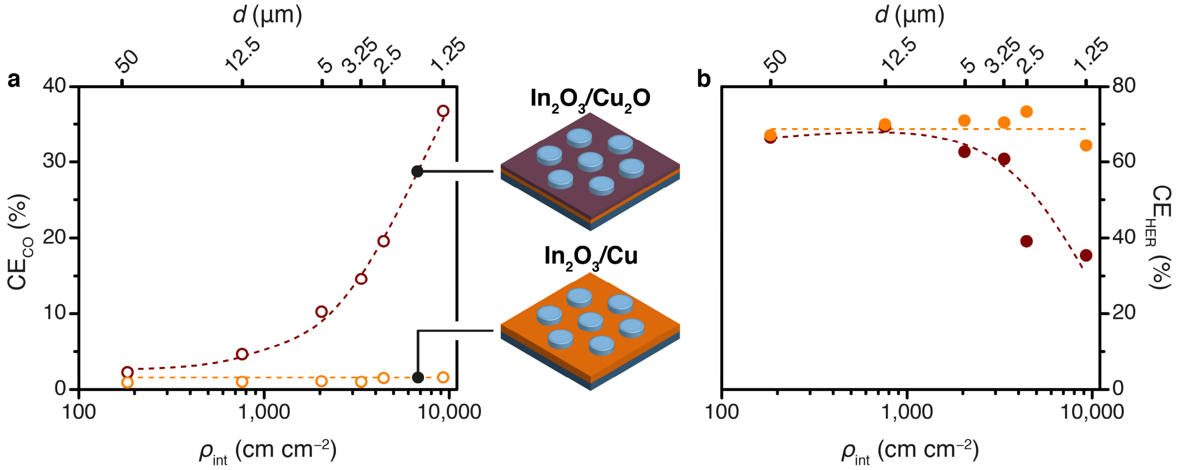

Supplementary Figure 5 | Current efficiency for CO and H_2 evolution over structured In_2O_3/Cu and In_2O_3/Cu_2O electrodes. Current efficiency for **a**, CO (CE_{CO}) and for **b**, H_2 (CE_{HER}) in eCO_2RR electrolyses as a function of the interfacial density (ρ_{int}) over In_2O_3/Cu and In_2O_3/Cu_2O structured electrodes. The corresponding island diameter (*d*) is also indicated. The electrolyses were carried out in CO_2-saturated 0.1 M $KHCO_3$ (pH 6.7) at −0.6 V vs. RHE. The dashed lines are added as a visual aid.

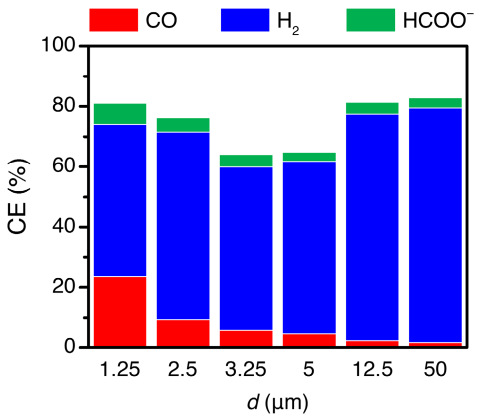

Supplementary Figure 6 | Current efficiency (CE) for different products in 5-minute eCO_2RR electrolyses over structured In_2O_3/Cu_2O electrodes. The gas-phase products (CO and H_2) were quantified by on-line GC measurements while $HCOO^-$ was determined by ^{1}H-NMR analysis of the catholyte and the anolyte following the reaction. Since the quantifiable products account only for 70-80% of the current efficiency, a comparatively large fraction of the charge transferred during the electrolyses likely corresponds to the reduction of In_2O_3 and Cu_2O. However, the re-oxidation of some $HCOO^-$ formed in the reaction following its migration to the anodic compartment cannot be discounted.

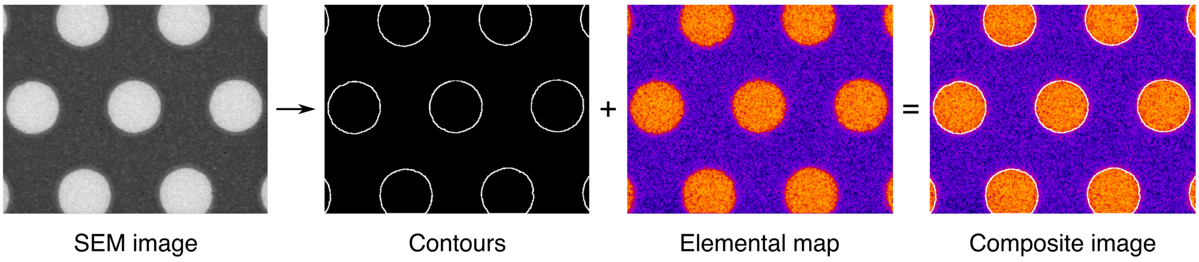

Supplementary Figure 7 | SEM image processing for the composition of elemental maps. The contours of the islands were extracted from backscattered electron micrographs and overlaid with EDX maps to facilitate the observation of the elemental distribution in the vicinity of the islands. The figure exemplifies the process for the In elemental distribution over an In_2O_3/Cu_2O electrode (d = 12.5 μm) after electrolysis.

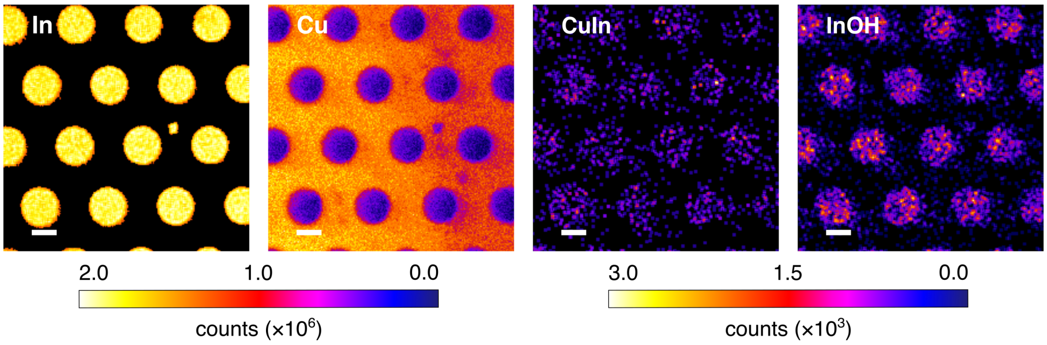

Supplementary Figure 8 | Chemical maps of In and Cu species acquired by ToF-SIMS from an In_2O_3/Cu_2O electrode following the reaction. In and Cu were preferentially detected over electrodes, as expected. The corresponding maps for $CuIn^+$ and $InOH^+$ confirm the formation of Cu-In intermetallic compound(s) over the islands, as expected from EDX chemical maps from cross-sections (**Fig. 5** of the main text), and indium hydroxide, likely as a result of exposure to air, respectively. Note the different count scales. Scale bars: 10 µm.

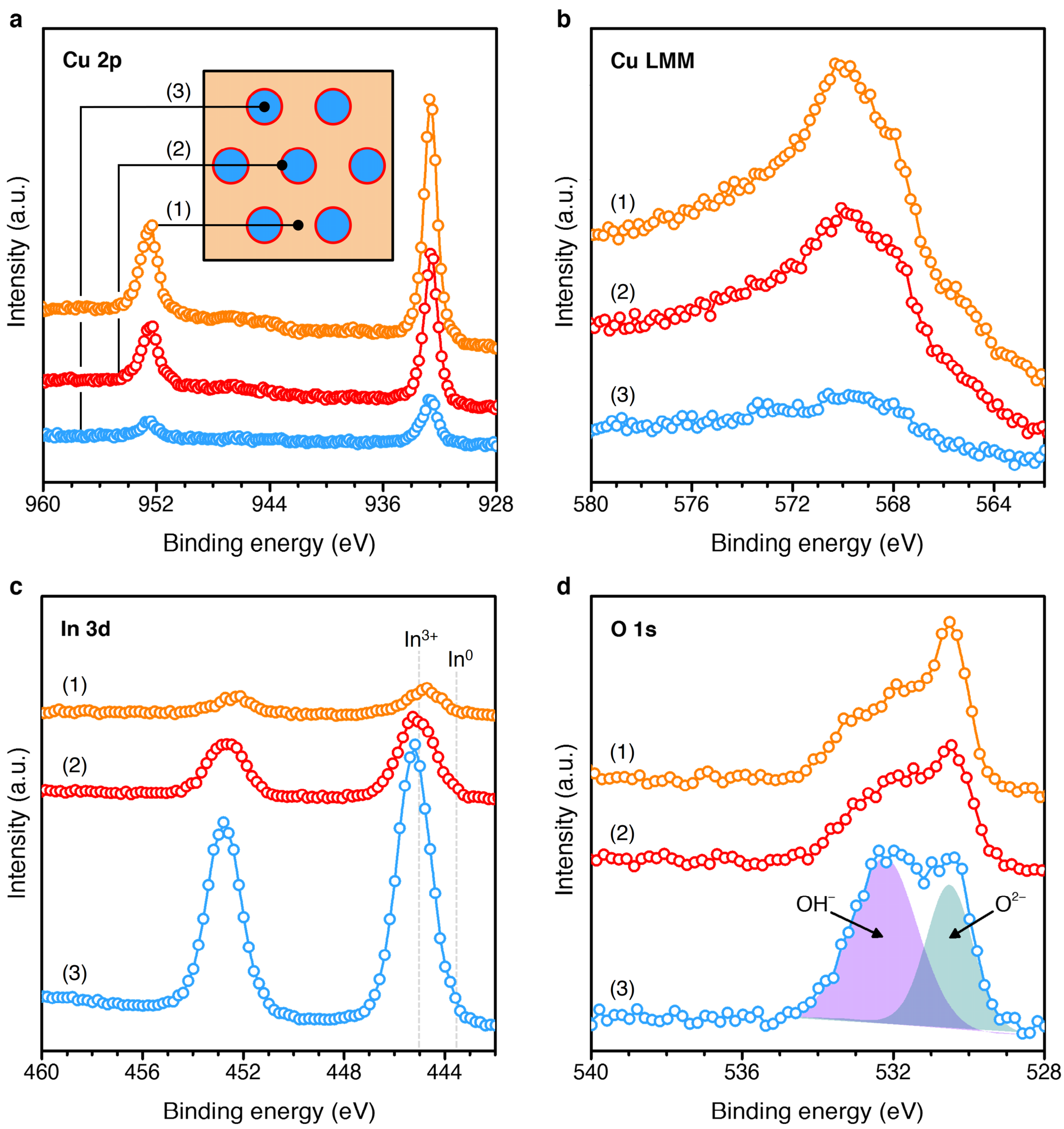

Supplementary Figure 9 | X-ray photoelectron spectroscopy analysis of a structured In_2O_3/Cu_2O electrode following the eCO_2RR electrolysis. XPS spectra around the **a**, Cu 2p; **b**, Cu LMM Auger; **c**, In 3d and **d**, O 1s regions of a used In_2O_3/Cu_2O electrode with large islands (d = 50 μm) acquired by centering the X-ray beam (with a nominal spot size of 20 μm) on different regions of the electrode indicated by the inset in **a**: (1) the substrate, (2) the interface between an island and the substrate (i.e., thus containing the halo region formed in situ, as described in the main text), and (3) the center of an island. No distinct spectral features from the halo region were distinguishable in the acquired spectra. The weak indium signal recorded from the bare substrate is likely caused by the long tail of the primary X-ray beam beyond its nominal size, resulting in limited lateral resolution. Peak positions for $In_2O_3/In(OH)_3$ (labelled as In^{3+} at 444.9 eV) and for metallic indium (443 eV) are indicated. The O 1s region shows an increased contribution from OH^- species of $In(OH)_3$ (peak at 531.9 eV, compared to O^{2-} species at 530.6 eV) when the X-ray beam was focused on the island. This likely corresponds to $In(OH)_3$ formed upon return of the electrode to open circuit potential and/or its subsequent exposure to air.

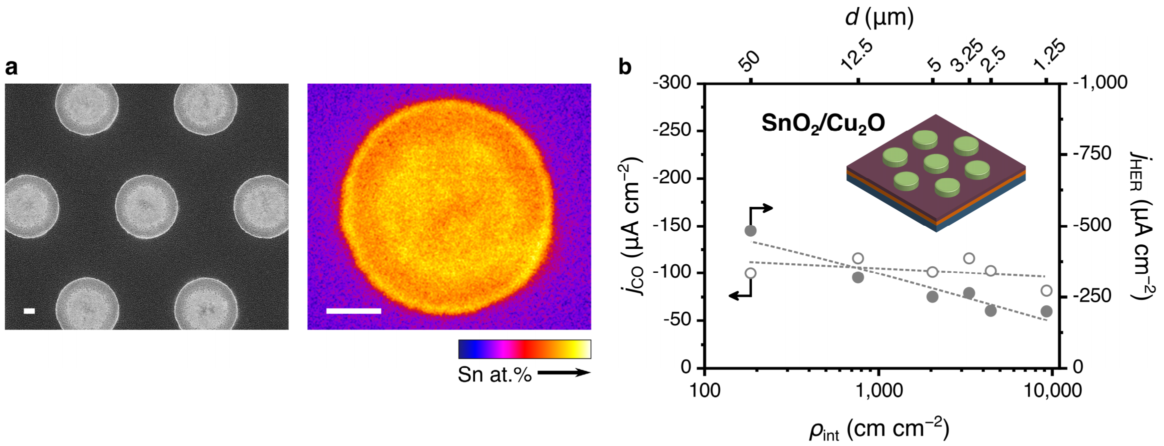

Supplementary Figure 10 | SEM micrograph obtained in backscattered mode and EDX chemical map of Sn, and catalytic activity for CO (j_{CO}) and H_2 evolution (j_{HER}) as a function of the interfacial density (ρ_{int}) over structured SnO_2/Cu_2O electrodes. a. Islands show an inner concentric pattern upon reaction associated to gradients in Sn concentration, as evidenced by EDX analysis. The presence of Sn several micrometers away from the islands indicate a fast Sn diffusion over the reduced Cu_2O substrate and thus, the likely modification of a large percentage of the Cu_2O substrate for most of the studied geometries. Scale bars: 2 µm. **b**. The catalytic activity shows no dependency on the interfacial density for the eCO_2RR and only a very mild one for the HER, in accordance with the visualization results. The electrolyses were carried out in CO_2-saturated 0.1 M $KHCO_3$ (pH 6.7) at −0.6 V vs. RHE. The dashed lines are added as a visual aid.
